# Regulation of Classical Cadherin Membrane Expression and F-Actin Assembly by Alpha-Catenins, during *Xenopus* Embryogenesis

**DOI:** 10.1371/journal.pone.0038756

**Published:** 2012-06-13

**Authors:** Sumeda Nandadasa, Qinghua Tao, Amanda Shoemaker, Sang-wook Cha, Christopher Wylie

**Affiliations:** 1 Division of Developmental Biology, Cincinnati Children’s Hospital Research Foundation, Cincinnati, Ohio, United States of America; 2 Graduate Program in Molecular and Developmental Biology, Cincinnati Children’s Hospital Research Foundation, Cincinnati, Ohio, United States of America; University of Birmingham, United Kingdom

## Abstract

Alpha (α)-E-catenin is a component of the cadherin complex, and has long been thought to provide a link between cell surface cadherins and the actin skeleton. More recently, it has also been implicated in mechano-sensing, and in the control of tissue size. Here we use the early *Xenopus* embryos to explore functional differences between two α-catenin family members, α-E- and α-N-catenin, and their interactions with the different classical cadherins that appear as tissues of the embryo become segregated from each other. We show that they play both cadherin-specific and context-specific roles in the emerging tissues of the embryo. α-E-catenin interacts with both C- and E-cadherin. It is specifically required for junctional localization of C-cadherin, but not of E-cadherin or N-cadherin at the neurula stage. α-N-cadherin interacts only with, and is specifically required for junctional localization of, N-cadherin. In addition, α -E-catenin is essential for normal tissue size control in the non-neural ectoderm, but not in the neural ectoderm or the blastula. We also show context specificity in cadherin/ α-catenin interactions. E-cadherin requires α-E-catenin for junctional localization in some tissues, but not in others, during early development. These specific functional cadherin/alpha-catenin interactions may explain the basis of cadherin specificity of actin assembly and morphogenetic movements seen previously in the neural and non-neural ectoderm.

## Introduction

The assembly of filamentous actin at the cell cortex has long been known to be essential for cell shape and motility. In embryos, actin-based changes in cell shape and motility are responsible for creating form, both of the whole embryo, and of its constituent organs. Since cells can migrate as individuals or can undergo complex morphogenetic migrations as cell masses, the relationship between cell-cell adhesion and cell migration must be tightly regulated. Cadherins mediate cell-cell adhesion and are also known to be the sites of membrane attachment of the actin skeleton [1], [2], as well as sites of actin assembly [3]–[6]. However, the mechanism regulating these events is still poorly understood.

The cadherin complex consists of the transmembrane glycoprotein cadherin, and cytoplasmic catenins that bind to the cadherin intracellular C-terminal tail. p120 catenin (p120ctn) is an armadillo repeat protein which binds to the juxta-membrane domain (JMD), close to the cell membrane, whilst two related proteins, β-catenin and plakoglobin, bind in a mutually exclusive fashion to the more C-terminal catenin-binding domain (CBD). β-catenin binds in turn to the vinculin homolog α-catenin, which has long been considered to link the cadherin/catenin complex to the actin skeleton through its C-terminal actin-binding domain. However, the precise relationship between α-catenin and actin at the cell membrane requires further examination, given evidence that α-catenin binding to β-catenin and actin are mutually exclusive in vitro [7], [8]. Recently, the protein Eplin/LIMA-1 has been identified as an intermediate binding partner between α-catenin and actin at adherens junctions [9]. In addition, proteins which mediate actin polymerization, including Arp2/3 [4], Formin [10], Ena/Vasp [11] and Cortactin [12] have also been found to be associated with the cadherin complex. Altogether, these data indicate that cadherin engagement both promotes actin assembly and physically links the cortical actin networks of adjacent cells.

We have previously used the early *Xenopus* embryo to study the role of cadherins in cortical actin assembly. At the blastula stage, all of the components of the cadherin complex, as well as the actin assembly proteins so far identified, are synthesized on mRNAs transcribed and stored in the oocyte, later to be replaced by zygotic mRNAs during gastrulation. Depletion of the stored maternal mRNA from the oocyte by microinjection of antisense oligodeoxynucleotides (AS oligos) into cultured oocytes, results in a corresponding depletion of the cognate protein until the zygotic mRNA is switched on [13], [14]. Using this method, we have previously shown that cortical actin assembly requires two types of G protein-coupled receptors (GPCRs); an orphan receptor Xflop [15], and receptors for the signaling ligand lysophosphatidic acid (LPA), [16]. These GPCRs control the amount of cell surface C-cadherin, the major transmembrane cadherin present in the early embryo [17]. Their action can be bypassed by increasing C-cadherin directly by mRNA injection, or decreasing it by mRNA depletion, which up-regulates and down-regulates respectively the density of cortical actin filaments. At later stages, when new tissue-restricted cadherins appear in different organ primordia, this basic mechanism appears to be retained. Depletion of N-cadherin in the neural plate, causes loss of actin assembly and abrogation of neurulation movements, whilst depletion of E-cadherin in the developing epidermis causes loss of actin assembly and abrogation of its normal spreading movements over the surface of the embryo [18].

These data indicate that cadherins do not simply link adhesion sites to a pre-assembled cortical actin cytoskeleton, but rather play a critical role in directing tissue-specific assembly of actin filaments at sites of cell-cell contact. This makes it extremely important to understand the mechanisms that regulate the amount and position of cadherin at the cell surface during morphogenesis, and the mechanism of actin assembly on cell surface cadherin.

In this paper, we show that the α-catenins play crucial, and cadherin-specific, roles in the expression of cadherins on the cell surface in the developing *Xenopus* embryo. α-E-catenin (αEC) is required both for junctional localization and the adhesive function of C-cadherin, both in the pre-gastrula embryo, and in the neural and non-neural ectoderm after gastrulation. In the post-gastrula ectoderm, α-N-catenin (αNC) is switched on in the neural ectoderm, whilst αEC continues to be expressed in both the neural and non-neural ectoderms. As these changes occur, the first signs of specificity appear in alpha-catenin-cadherin interactions. N-cadherin interacts only with αNC, and requires αNC, but not αEC for junctional localization and actin assembly. C-cadherin interacts with αEC but not αNC, and requires αNC for junctional localization and actin assembly at all stages and tissues. E-cadherin is more complex. At the neurula stage, it’s expression on the junctions in the non-neural ectoderm is αEC-independent. However, at the late blastula stage, it shows a context-dependent requirement for αEC. In the polarized outer cells of the blastula it’s cell surface expression is αEC-independent, whereas in the non-polarized inner cells, the opposite is the case. These data help explain the mechanism of the cadherin-based specificity of actin assembly and morphogenetic movements shown previously for the neural and non-neural ectoderms.

## Results

### α-catenin Function in the Blastula

**Figure 1:**
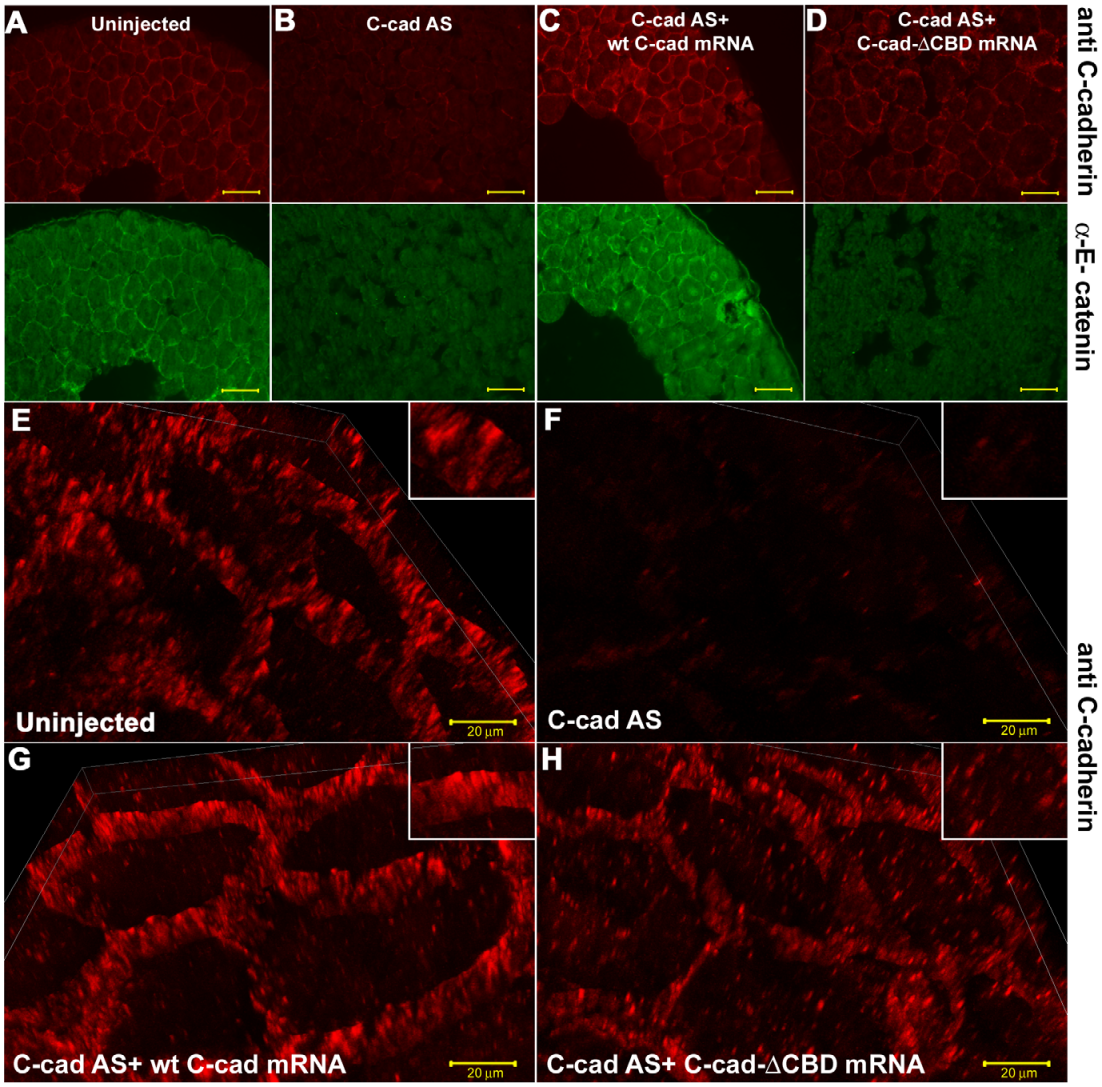
The C-cadhering catenin binding domain (CBD) is required for cell adhesion . (A–D ) Immunostaining for C-cadherin (upper panels) and αEC (lower panels) in paraffin sections of stage 9 embryos, showing the non-rescue of cell adhesion in the absence of the CBD. (**E–H**) 3D projections of high magnification, and high resolution confocal images showing the localization of C-cadherin protein. Scale bars for A-D, 50 µM, E-H, 20 µM.

The function of αEC at the late blastula stage was investigated first by comparing the effects of replacing C-cadherin, the major cadherin at this early stage, with mutant forms either lacking the CBD domain (C-cad ΔCBD), and thus unable to bind αEC, or with the CBD domain replaced by the C-terminus of αEC. This was achieved by depleting the endogenous C-cadherin mRNA using an antisense oligo as described previously [13] followed by injection of the appropriate mRNA into cultured oocytes before fertilization. **Fig. 1A, B, C, D** show sections of late blastulae (St.9) stained for C-cadherin (red, upper panels) and αEC (green, lower panels) after no treatment (**Fig. 1A**), depletion of C-cadherin (**Fig. 1B**), depletion of C-cadherin + 300 pg wild type C-cadherin mRNA (**Fig. 1C**), and depletion of C-cadherin + 300 pg C-cad ΔCBD (**Fig. 1D**). The degree of cell dissociation can be seen in the lower panels, where spaces between the cells can be more easily seen (dark areas) due to the background staining of the cell contents. Depletion of C-cadherin mRNA caused the loss of C-cadherin protein from the cell surface and cell dissociation. It’s replacement by wild-type C-cadherin replaced cell surface C-cadherin protein and rescued cell adhesion. However, replacement by C-cad ΔCBD did not rescue cell adhesion. Although some of the mutant protein was expressed on the surface (upper panel in **Fig. 1D** **),** it was not associated with αEC (as expected, since this lacks the CBD domain), and was non-functional in cell adhesion. The result is shown more clearly in **Fig. 1E – H**, in which sections stained for C-cadherin have been observed. The high magnification 3D projections show that C-cadherin is concentrated in plaques on the cell surfaces in untreated embryos (**Fig. 1E**), which are lost after C-cadherin depletion (**Fig. 1F**), and regained at higher density after replacement by wild-type C-cadherin (**Fig. 1G**). When replaced by C-cad ΔCBD, however, less of the mutant protein was expressed on the surface, and more remained trapped in the cytoplasm (**Fig. 1H**).

**Figure 2:**
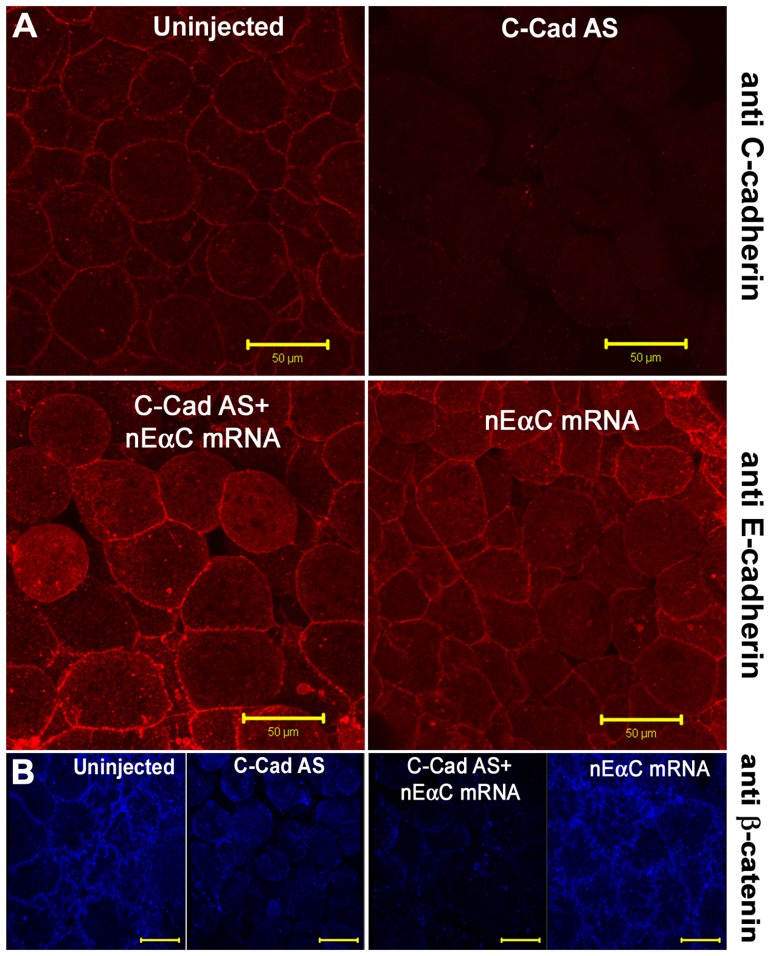
C-terminus of α-E-catenin is sufficient to drive cadherin membrane expression. **(A**) Immunostaining for C-cadherin (upper panels) and E-cadherin extracellular domain (lower panels) in animal caps, showing the rescue of cell adhesion and the expression of nEαC in cell junctions. (**B**) Immunostaining for β-catenin showing the junctional localization of nEαC is not through β-catenin. Scale bars for A-B, 50 µM.

We then carried out a similar experiment in which C-cadherin was replaced by a construct in which the CBD domain of mouse E-cadherin was replaced by the C-terminal half of αEC (nEαC).In contrast to the C-cad ΔCBD protein, nEαC was efficiently expressed on the cell surface and rescued cell adhesion (**Fig. 2A**). In these rescued embryos, there was no cell surface staining with anti-β−catenin antibody, indicating that the rescue was by the nEαC protein and not by residual wild-type C-cadherin protein (**Fig. 2B**).These data suggest that the primary function of the CBD is to bind αEC, and that this binding to C-cadherin is essential for its normal expression and/or stability on the cell surface, and for its adhesive function.

**Figure 3:**
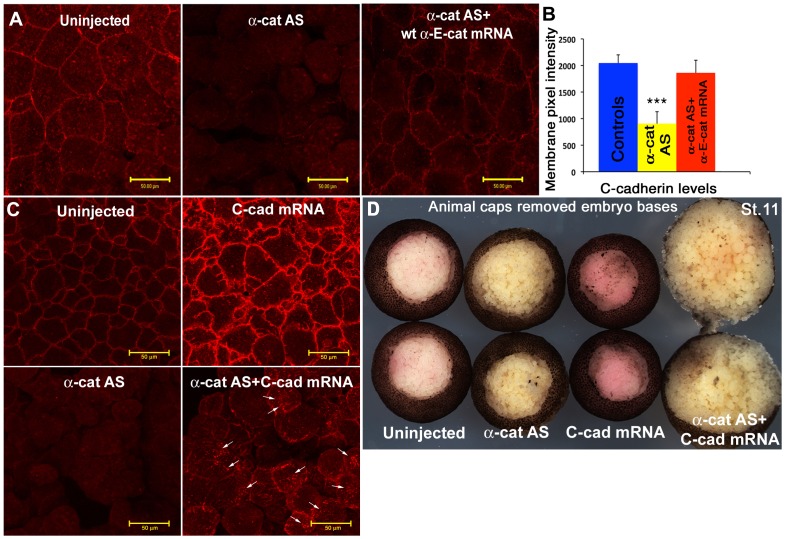
α-E-catenin is required for C-cadherin expression on the cell surface in the blastula. (**A**) Immunostaining of animal caps stained for C-cadherin, showing that the loss of C-cadherin junctional localization caused by αEC depletion can be rescued by the expression of wild type αEC mRNA.(**B**) Quantification of cell junction-localized C-cadherin levels. (**C**) Immunostaining of animal caps stained for C-cadherin, showing that the loss of C-cadherin junctional localization caused by αEC depletion cannot be fully rescued by the injection of C-cadherin mRNA.(**D**) Wound healing assay of embryos in which the animal caps have been dissected out and healed for 1 hr in 1×MMR, showing the increased negative effect of both α-catenin depletion and C-cadherin over-expression. Scale bars in A, C 50 µM.

To test this further, we depleted early embryonic αEC directly, using the antisense oligo described previously [19], or a novel translation-blocking morpholino oligo (αEC-MO) complementary to the translation start site of *Xenopus laevis* αEC. **Fig. 3A** shows the blastocoelic surfaces of whole animal caps stained for C-cadherin from embryos that were untreated, αEC-depleted, or αEC-depleted then rescued with full-length human αEC mRNA. Pixel intensity measurements using the Zeiss LSM software were used to quantitate the amount of C-cadherin antibody staining (**Fig. 3B**). Embryos depleted of αEC were also injected with full-length C-cadherin mRNA. In these embryos, some of the full-length protein was expressed on the cell membrane, but this was reduced compared to controls, and it did not rescue the dissociation effect of αEC depletion (**Fig. 3C, D**).

Thus the two approaches; replacement of C-cadherin with a mutant that lacked the αEC binding domain, or reducing directly the αEC protein levels, both gave the same result; that αEC is required for cell adhesion because it controls the expression levels of C-cadherin in the adherens junctions of the blastula cells.

### Alpha Catenin Function in the Post-gastrula Ectoderm

**Figure 4:**
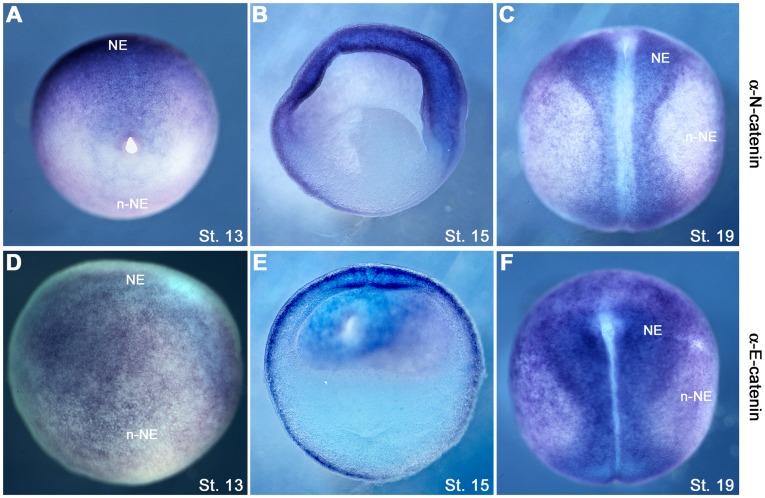
The distribution of α-N-catenin and α-E-catenin mRNAs in the neural and non-neural ectoderm. (**A-C**) αNC *In-situ* staining of st.13–19 embryos (**D-F**) αEC In-situ staining of st.13–19 embryos. NE (Neural Ectoderm), n-NE (non-Neural Ectoderm).

The ectoderm was used as a model in this study, since its cells can be directly visualized on the outside of the embryo, and it differentiates into two components, the neural and non-neural ectoderm, each with a distinct expression pattern of cadherins and α-catenins. The non-neural ectoderm expresses C- and E-cadherins, whilst the neural ectoderm expresses C- and N-cadherins, each with characteristic sub-cellular distributions [18]. The neural ectoderm expresses αNC [20]–[22] and αEC [23], whilst the non-neural (epidermal) ectoderm expresses only αEC [23]. In-situ hybridization of *Xenopus* whole mounts confirmed the previously reported patterns of expression of these two alpha catenins in other species (**Fig. 4** **A, B, C, D, E, F**).

**Figure 5:**
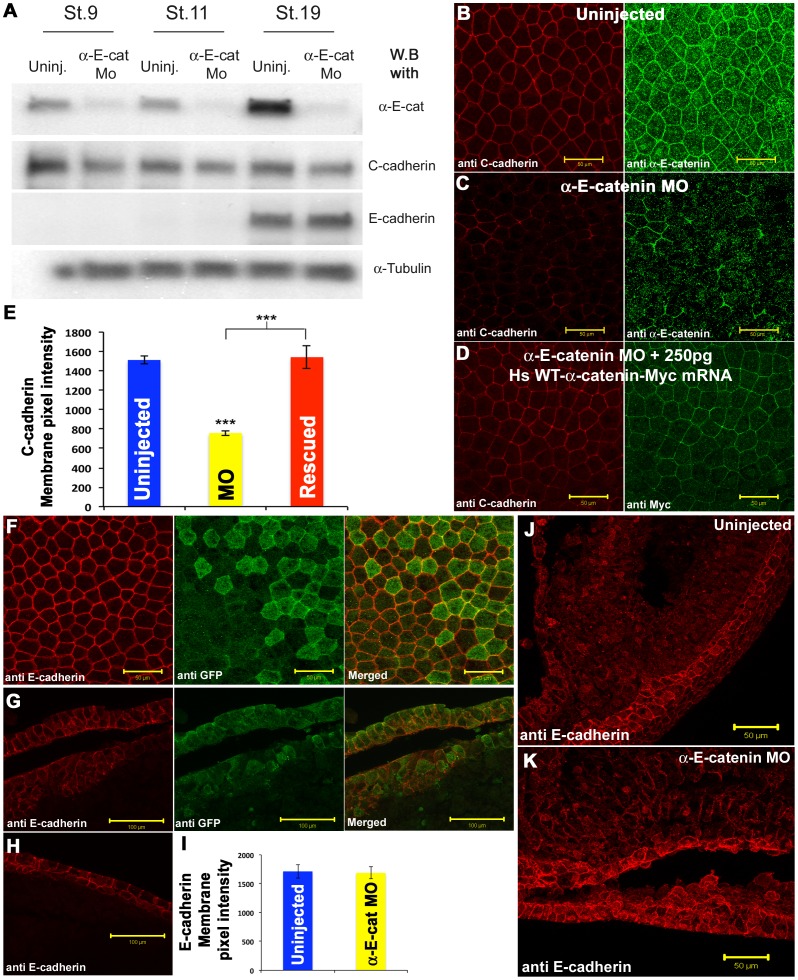
Antisense morpholino mediated depletion of α-E-catenin in the non-Neural Ectoderm. (**A**) Western blot showing the level of αEC, C-cadherin, and E-cadherin protein levels in uninjected and αEC-MO-injected embryos at st.9,11, and 19.(**B-D**) Confocal projections of the non-neural ectoderm of wild type (**B**) or αEC-depleted (**C**) st. 19 embryos, stained for C-cadherin (Red) and αEC (Green), and rescue of C-cadherin (Red) junctional localization by the introduction of Myc-tagged (green), human wild type αEC (**D**). (**E**) Quantification of pixel intensity for C-cadherin levels. (**F**) E-cadherin (Red) junctional localization in αEC-depleted cells (Green, GFP) in the non-neural ectoderm. (**G-H**) Cross sections of αEC-depleted (**G**), or uninjected (**H**) embryos, stained for E-cadherin (Red) and GFP (green). (**I**) Quantification of pixel intensity for E-cadherin levels. (**J-K**) Wholemount vibratome sections stained for E-cadherin in wildtype (**J**) or αEC-depleted (**K**) embryos. Scale bars in B-D, F, J-K 50 µM, G-H 100 µM.

To test the roles of the two α-catenins at post-gastula stages, we injected morpholino oligos complementary to the translation start site of each mRNA into individual blastomeres at the 8-cell stage whose descendants would either enter the neural (single dorsal animal blastomere) or non-neural (single ventral animal blastomere) ectoderm respectively. The efficiency of the αEC-MO is shown in **Fig. 5A**. The antibody used to detect αNC did not cross react on western blots, so its efficiency was assayed by immunocytochemistry (see below). **Figure 5B** shows an *en face* view of the non-neural ectoderm stained for C-cadherin (red) and αEC (green). In clones of cells depleted of αEC (**Fig. 5C**), C-cadherin expression on the cell surface was lost. When human alpha-E-catenin mRNA, which is not recognized by the αEC-MO, was injected into the same blastomere immediately after the MO, the effect was rescued (**Fig. 5D**). Thus, αEC has the same function in controlling cell surface expression of C-cadherin as seen in the blastula. However, the effect of αEC depletion on E-cadherin expression was completely different. *En face* views of αEC-depleted clones showed the expression of E-cadherin on junctions to be unaffected (**Fig. 5F**). Staining of E-cadherin was indistinguishable in αEC-depleted cells (green) and untreated cells. However, examination of transverse sections revealed that the non-neural ectoderm was multi-layered in αEC-depleted regions (**Fig. 5G**) compared to the untreated non-neural ectoderm from the same embryo (**Fig. 5H**), which shows the usual two cell layers. This multi-layered arrangement resembled the cyst-like structures described in mice lacking αEC in the epidermis [24]–[26]. When αEC was depleted globally by injection of the αEC-MO into both cells at the 2-cell stage, there was a loss of αEC throughout the embryo, but high levels of E-cadherin remained present in the non-neural ectoderm, and there was some up-regulation of expression of E-cadherin in deeper cells in the embryo (**Fig. 5J and K**). Interestingly, western blotting data showed that the overall levels of C-cadherin protein were only modestly reduced after αEC depletion (**Fig. 5A**). To visualize the formation of cyst-like structures in αEC-depleted non-neural ectoderm, we depleted αEC in one side of the embryo and made time lapse movies. In these embryos, large cyst-like structures started to appear immediately after the neural folds closed, when the non-neural ectoderm cells normally start to intercalate and spread by epiboly **(Movie S1)**.

**Figure 6:**
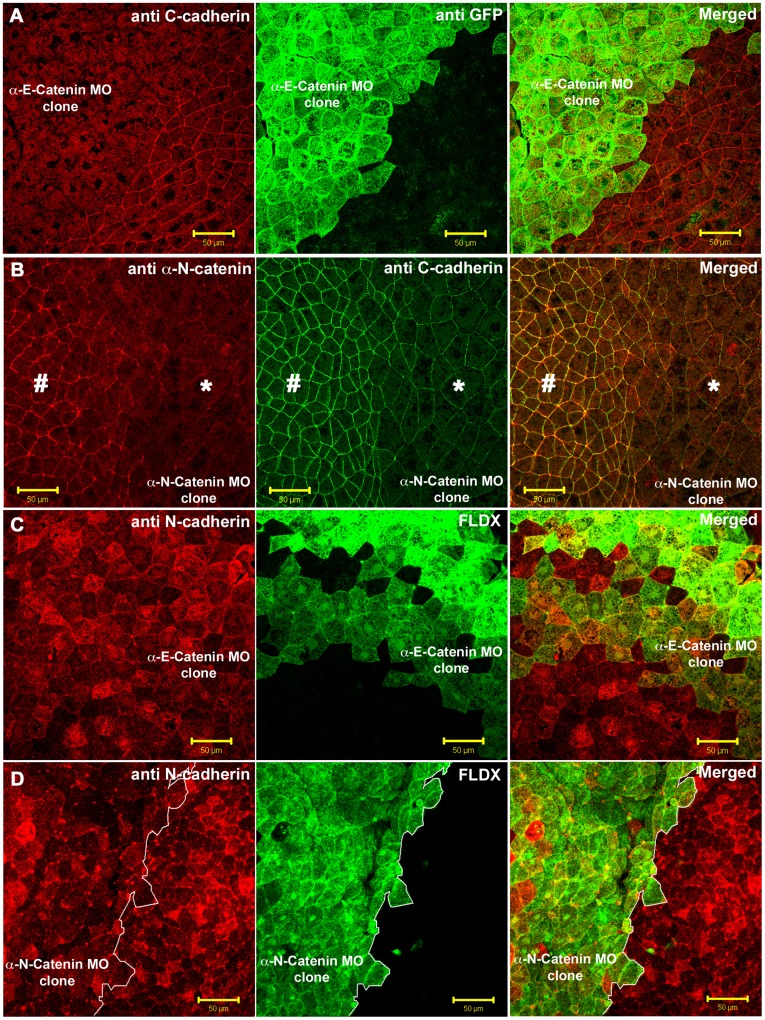
Depletion of α-E-catenin and α-N-catenin in the neural ectoderm. (**A**) Neural plates containing αEC-depleted clones (green) stained for C-cadherin (Red) and GFP (Green). (**B**) Neural plates containing αNC-depleted clones (morpholino-injected side marked by *, and uninjected side marked by # ), stained for αNC (Red) and C-cadherin (Green). (**C**) αEC-depleted neural plate cells (Green, FLDX) stained for N-cadherin (Red). (**D**) αNC-depleted neural plate cells (Green, FLDX) stained for N-cadherin (Red). Scale bars in **A-D**, 50 µM.

Next we depleted the α-catenins in the neural ectoderm. Clones of cells depleted of αEC, αNC, or both, were generated on one side of the neural ectoderm by injection of MO, together with a lineage tracer, into one dorsal animal cell at the 8-cell stage. As it did in the non-neural ectoderm, depletion of αEC caused a dramatic decrease in junctional localization of C-cadherin (**Fig. 6A**, the αEC-depleted cells are green). **Fig. 6B** shows a clone of αNC-depleted cells. The tissue was stained with an antibody against αNC, and shows the degree of depletion in injected, compared to uninjected cells in the same neural plate. The neighboring panel shows the same cells stained for C-cadherin. The staining was reduced, but not completely absent compared to αEC-depletion (compare with **Fig. 6A**). The surface areas of the apices of the neural plate cells were increased in αNC depleted cells. This effect was also seen after depletion of N-cadherin, and is caused by the loss of the F-actin in the apical regions of the neural plate cells [18]. It is hard to know whether the difference is due to loss of the concentration effect on C-cadherin caused by the lack of apical constriction of the neural plate cells, or by an actual decrease in overall C-cadherin levels on the cell surface. However, unlike the effect of αEC depletion, a significant amount of C-cadherin remained in the cell junctions after αNC depletion. The opposite was true for N-cadherin. Depletion of αNC, but not αEC, caused complete loss of N-cadherin from the cell surface in the neural ectoderm (**Fig. 6C and D**). **Fig. 6C** shows a clone of αEC-depleted cells (green) in the neural plate, stained for N-cadherin (red). There was no reduction of N-cadherin expression. **Fig. 6D** shows a clone of αNC-depleted cells (green) in the neural plate. N-cadherin was completely lost from the cell surface. In chick neural epithelium, the depletion of α-N-catenin has also been shown to cause a loss of N-cadherin expression from the cell surface [27].

In summary, αEC is required for C-cadherin expression on the cell surface in both the neural and non-neural ectoderm, but is not required for E-cadherin expression in the non-neural ectoderm, or N-cadherin expression in the neural ectoderm. αNC is essential for N-cadherin cell membrane expression in the neural ectoderm, but not required for C-cadherin expression. These data indicate that there is considerable specificity of function of the α-catenins in the post-gastrula embryo.

**Figure 7:**
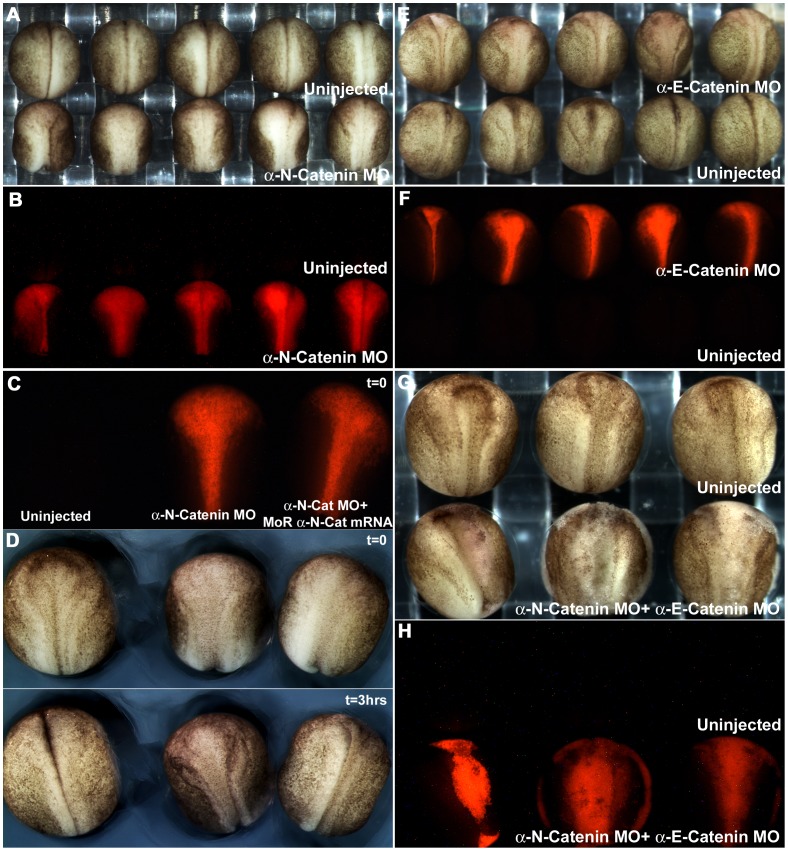
α-N-catenin is critical for the normal morphogenesis of the neural tube. (**A**) Robust open neural plate phenotype observed in αNC morpholino-injected embryos. (**B**) RLDX fluorescent signal marking the descendents of αNC morpholino-injected cells of embryos shown in (**A**). (**C**) RLDX fluorescent signal marking the descendents of αNC morpholino and rescued mRNA-injected cells, for embryos shown in (**D,** first frame). (**D**) First and last frames of a timelapse movie taken at 5 minute intervals, showing the rescue of neurulation movements by sequentially injecting αNC mRNA into morpholino-injected cells. (**E**) Delay in neurulation movements observed in αEC-depleted neural plates. (**F**) RLDX fluorescent signal marking the descendents of αEC morpholino-injected cells of embryos shown in (**E**). (**G**) Embryos depleted of both αEC and αNC, showing cell dissociation in their neural plates. (**H**) RLDX fluorescent signal marking the descendents of αEC and αNC morpholino-injected cells for embryos shown in (**G**).

Previous work showed that loss of E-cadherin in the non-neural ectoderm, or N-cadherin in the neural ectoderm, caused the loss of F-actin in the cortical cytoplasm, and abrogation of the morphogenetic movements of these tissues [18]. To assay the functions of the α-catenins in these effects, we first assayed the result of depleting αEC and αNC in the non-neural and neural ectoderms respectively, and depletion of both in the neural ectoderm. The effect of αEC depletion in the non-neural ectoderm, the formation of large cysts, has already been noted (**Movie S1**). Depletion of αNC in both sides of the neural plate, by injection of both dorsal animal cells at the 8-cell stage, caused an effect identical to that caused by the depletion of N-cadherin, the failure of the neural plate to invaginate (**Fig. 7A and B**). This was rescued by the subsequent injection of morpholino-resistant form of αNC mRNA into each morpholino-injected cell (**Fig. 7C and D**). For the time-lapse movie showing the rescue of the morphogenetic movements, see **Movie S2**. αEC depletion in the neural plate caused a similar, but less severe phenotype (**Fig. 7E and F**), and these embryos did eventually close their neural tubes, but were significantly delayed. Depletion of both α-catenins simultaneously in the neural plate caused complete cell dissociation of the neural plate during neurulation (**Fig. 7G and H**).

**Figure 8:**
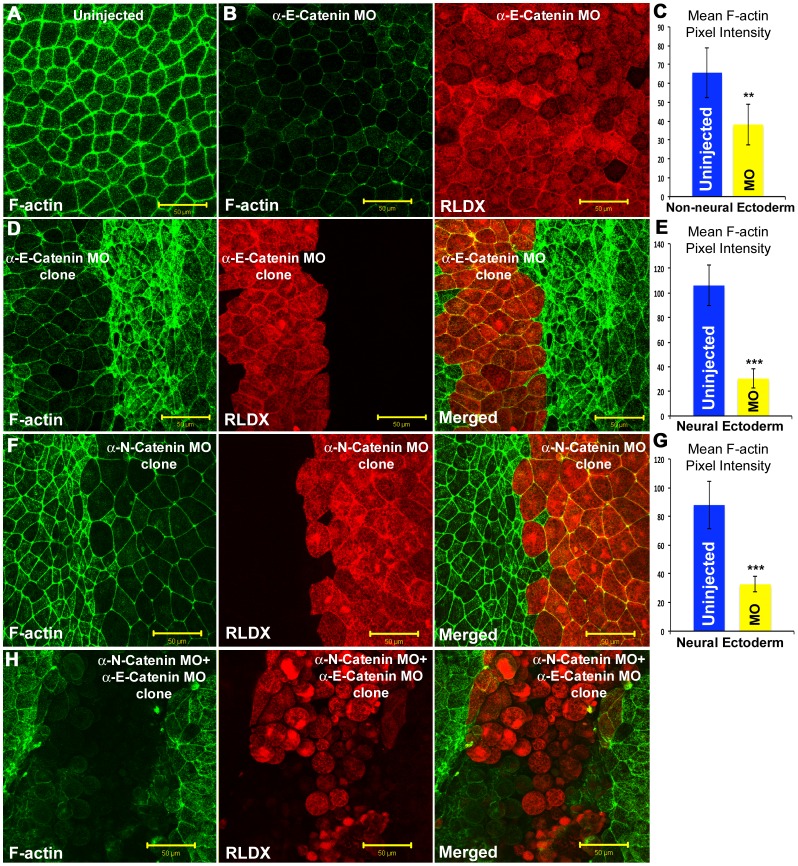
Cortical F-actin staining in alpha catenin-depleted neural and non-neural ectodermal cells. (**A**) Alexa-488 phalloidin staining for F-actin (green) in uninjected non-neural ectoderm cells. (**B**) αEC morpholino-injected non-neural ectoderm cells (Red, RLDX) showing the loss of F-actin (green). (**C**) Mean F-actin pixel intensity quantitation in uninjected and αEC-depleted non-neural ectoderm cells. (**D**) F-actin staining (green) in αEC-depleted neural ectoderm cells (Red). (**E**) Mean F-actin pixel intensity quantitation in uninjected and αEC-depleted neural ectoderm cells. (**F**) F-actin staining (green) in αNC-depleted neural ectoderm cells (Red). (**G**) Mean F-actin pixel intensity quantitation in uninjected and αNC-depleted neural ectoderm cells. (H) Neural plates stained for F-actin (green) in αEC, and αNC-depleted neural ectoderm cells (red). Scale bars, 50 µM.

In light of these phenotypic effects, we compared the amount of F-actin after αEC and αNC depletion in the neural and non-neural ectoderms, using Alexa488-phalloidin to stain for F-actin. Depleted clones of cells were identified using the lineage tracer RLDX, co-injected with the morpholinos into single cells at the 8-cell stage. Pixel intensity measurements were used to quantitate F-actin levels. αEC depletion in the non-neural ectoderm caused a dramatic loss of F-actin throughout the cells (**Fig. 8A, B, C**), even though E-cadherin junctional expression was unaltered. Depletion of either αEC or αNC in the neural plate caused a decrease in F-actin (**Fig. 8D, E, F, G**). Depletion of both α-catenins in the neural plate caused complete loss of F-actin and cell dissociation.

**Figure 9:**
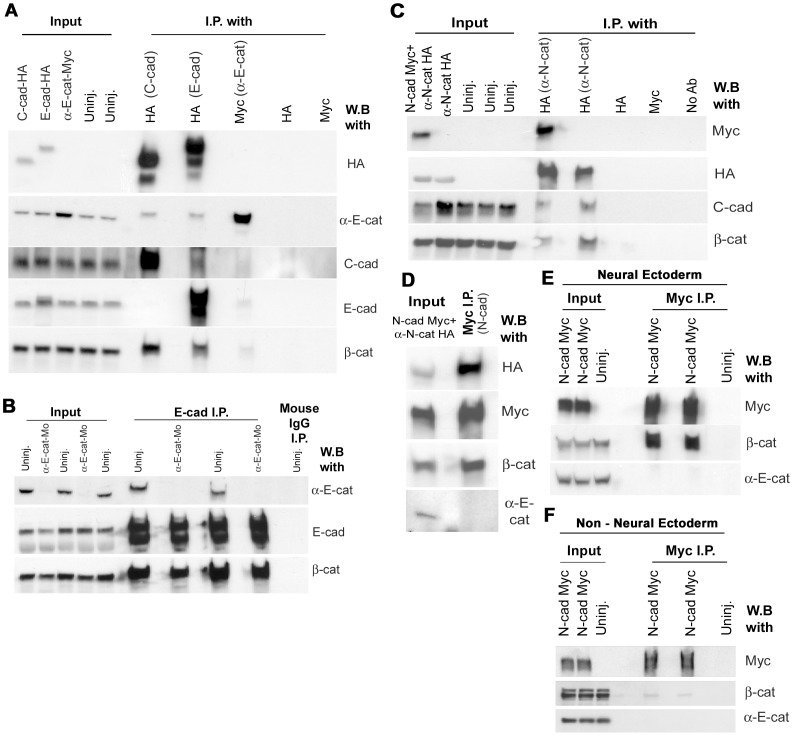
Co-Immunoprecipitations of cadherin-catenin complexes. (**A**) Western blot data of immunoprecipitations of the C-, and E-, cadherin complexes, using HA-tagged C-, and E-, cadherin, and Myc-tagged α-E-catenin, expressed in the non-neural ectoderm. (**B**) Western blot data of duplicate E-cadherin immunoprecipitations on wild type or αEC-depleted embryos. (**C**) Western blot data of immunoprecipitating N-cadherin-Myc or αNC-HA expressed in the neural ectoderm. (**D**) Sequential immunoprecipitation of N-cadherin-Myc from the lystae of N-cadherin-Myc + αNC-HA mRNA-injected embryos from (**C**). (**E**) Replicate immunoprecipitations of N-cadherin-Myc in embryos expressing N-cadherin-Myc in their neural ectoderms. (**F**) Replicate immunoprecipitations of N-cadherin-Myc in embryos expressing N-cadherin-Myc in their non neural ectoderms.

These data suggest that α-catenins have specificity in their binding to cadherin complexes containing different cadherins. To assay the degree to which each cadherin interacts with the other cadherins, and with the two α-catenins, we carried out co-immunoprecipation experiments. First, HA-tagged C-cadherin or E-cadherin was expressed in the non-neural ectoderm by injection at the 8-cell stage. Immunoprecipitation with anti-HA of lysates from the two batches of embryos showed that αEC was co-immunoprecipated by both C- and E- cadherin, indicating that αEC binds to both cadherins in the non-neural ectoderm (**Fig. 9A**). This result was also found in the complementary experiment of expressing myc-tagged αEC into the non-neural ectoderm and immunoprecipitating with anti-myc. Although this antibody did not immunoprecipate efficiently, both cadherins were present in the precipitate at low levels (**Fig. 9A**). Interestingly in this experiment, neither E- or C-cadherin interacted strongly, if at all, with the other, suggesting the possibility that they may be in different junctional complexes in the non-neural ectoderm (**Fig. 9A**). These data also suggest that the continued presence of E-cadherin in cell junctions in αEC-depleted embryos is not due simply to the fact that it doesn’t bind αEC. To eliminate the possibility that the membrane-localized E-cadherin seen in αEC-depleted embryos was due to binding of residual αEC present, we immunoprecipated E-cadherin from lysates of embryos injected with αEC-MO (**Fig. 9B**). No αEC was found in immunoprecipates, although β-catenin was present, indicating that E-cadherin is not expressed on the surface through binding to residual αEC in these experiments.

Next, we assayed for interactions in the neural ectoderm. Either Myc-tagged N-cadherin and HA-tagged αNC together, or HA-tagged αNC alone, were expressed in the neural ectoderm by injection of mRNAs into both dorsal animal cells at the 8-cell stage. N-cadherin and αNC each co-immunoprecipated the other, and αNC co-immunoprecipated C-cadherin (**Fig. 9C**). However, N-cadherin did not co-immunoprecipate any endogenous αEC, even though it was abundant in the input lysate (**Fig. 9D**). To eliminate the possibility that overexpression of tagged αNC in this experiment may have swamped out any possibility of interaction between N-cadherin and endogenous αEC, we repeated the experiment by expressing Myc-tagged N-cadherin only in the neural ectoderm. Once again, no endogenous αEC was co-immunoprecipated by anti-myc, although an abundant amount of β-catenin was co-immunprecipated (**Fig. 9E**). These data show that N-cadherin interacts specifically with αNC, and not αEC, in the neural ectoderm. This explains the fact that N-cadherin on the cell surface is abrogated by depletion of αNC, but not αEC (**Fig. 7C and D**). They also show that C-cadherin reacts with both alpha catenins, which may explain the partial reduction in C-cadherin levels on the cell surface when αNC is depleted (**Fig. 7B**). In order to test whether N-cadherin can interact with αEC in the absence of αNC, we ectopically expressed Myc-tagged N-cadherin specifically in the non neural ectoderm and immunoprecipitated using an antibody against myc. In contrast to the neural ectoderm, only a sparse amount of beta catenin and virtually no αEC was co-immunoprecipitated by N-cadherin-Myc (**Fig. 9F**). These data suggest that N-cadherin may be utilizing a different set of catenins and binding partners when expressed ectopically in the presumptive epidermis.

**Figure 10:**
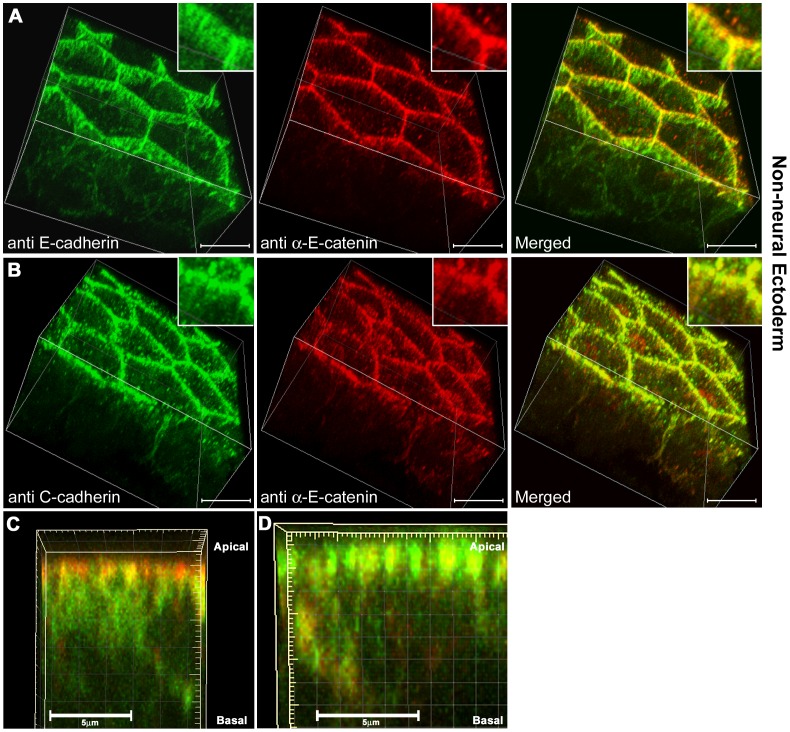
Three-dimensional projections of high resolution confocal images of cleared non-neural ectodermal cells. (**A**) E-cadherin (green) and αEC (red) staining in wholemount, cleared, non-neural ectoderm samples imaged at 100× in nyquist limit and using a 2× zoom. (**B**) C-cadherin (green) and αEC (red) staining.(**C**) Side view projection of a 3D image stack from a single cell-cell contact membrane, E-cadherin (green) and αEC (red). (**D**) Side view projection of a 3D image stack from a single cell-cell contact membrane, C-cadherin (green) and αEC (red). Scale bars in A-B, 15 µM, C-D, 5 µM.

The most parsimonious explanation of the immunoprecipitation and depletion data in the non-neural ectoderm would be that there are different cadherin-containing junctions present, some containing C-cadherin but not E-cadherin (because they do not co-immunoprecipate), and that either E-cadherin-containing junctions do not bind αEC, or do not require binding (because αEC depletion did not cause a loss of E-cadherin from cell junctions). To test these possibilities, high resolution confocal imaging was carried out on cleared ventral ectoderm tissues co-stained for E-cadherin and αEC (**Fig. 10A**), or C-cadherin and αEC (**Fig. 10B**). The results supported this hypothesis. First, E-cadherin (green in **Fig. 10A** **)** was found to be distributed throughout the baso-lateral cell surfaces, but concentrated in a sub-apical belt of junctions. αEC in the same fields of view (red in **Fig. 10A**) was concentrated apically, and merged images showed that E-cadherin and αEC only partially overlapped. Around the apical surface of the cells, areas of red but no green could be seen, in addition to yellow areas where the staining overlapped. Baso-laterally, most of the E-cadherin-positive areas did not overlap with αEC staining as shown by the 3D side-view of a single cell surface (**Fig. 10C**). In contrast, there was almost complete overlap of the C-cadherin and αEC staining, which was confined to the more apical regions of the cells (**Fig. 10B and D**). Although these images are at the limits of resolution of the confocal microscope, there is a clear difference between the distributions of the cadherins themselves, and in their interactions with αEC. To view the 3D projections see **movies S3** and **S4**.

**Figure 11:**
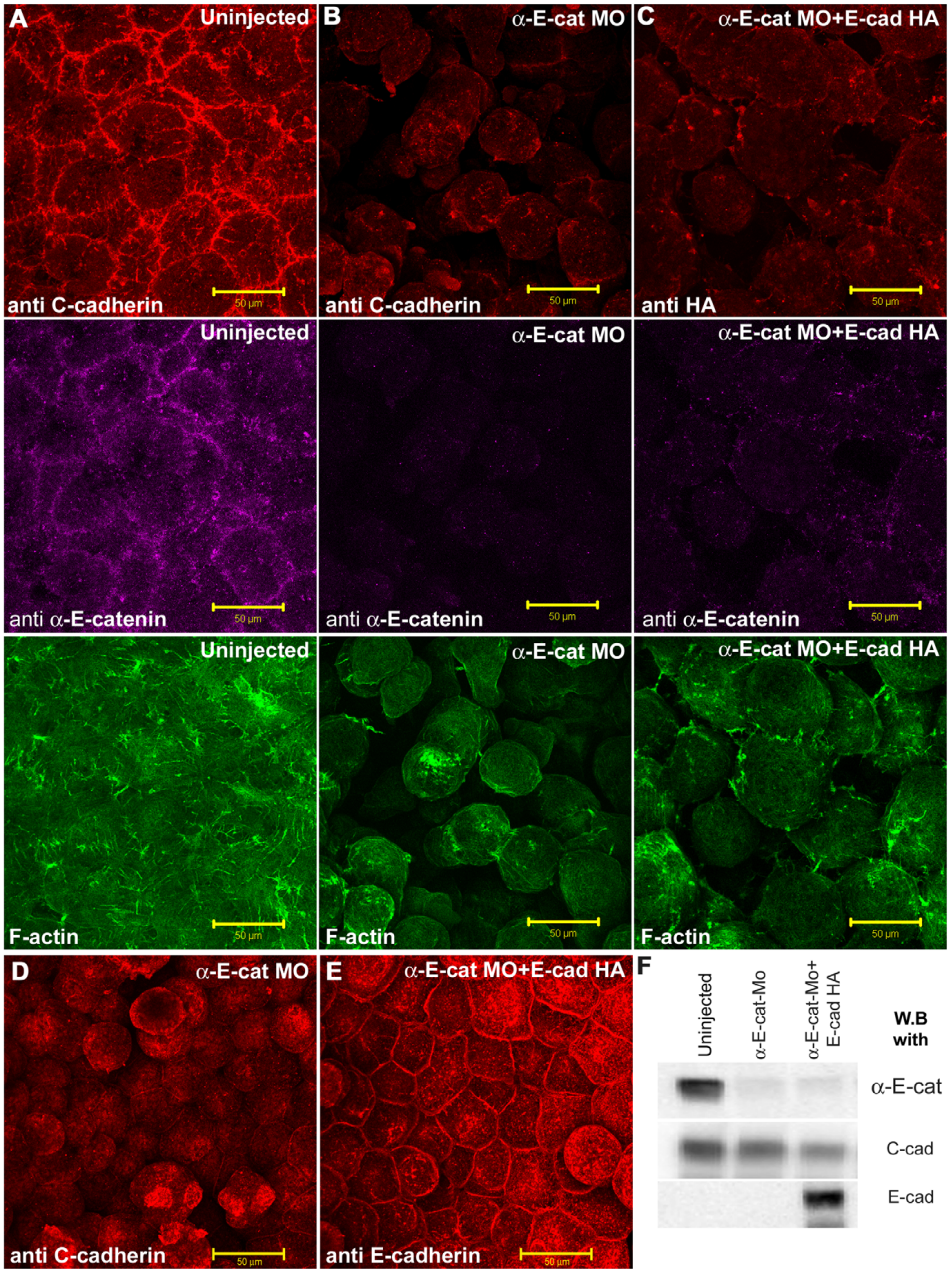
E-cadherin requires α-E-catenin in the non-polarized blastocelic roof cells, but not in the polarized superficial animal cap cells for junctional localization and cell adhesion. (A-C) Blastocelic roof cells of st. 9 animal caps stained for C-cadherin (red in A and B), or E-cadherin-HA (red in C), αEC (purple), and F-actin (green) in uninjected (A), αEC-depleted (B), or αEC + E-cadherin-HA mRNA injected (C) embryos. (D-E) Superficial cells imaged from the blastocelic cavity in animal caps where all the deeper cells have fallen off, stained for C-cadherin in (D), or E-cadherin (E), in αEC depleted (D), or αEC depleted + E-cadherin-HA mRNA-injected (E) embryos. (F) Western blot data showing the depletion of αEC protein and the expression of C-, and E-, cadherin protein levels in the experiment. Scale bars, 50 µM.

These data suggest that the dependence on αEC binding for E-cadherin junctional expression may be context-dependent in the non-neural ectoderm. In some junctions the two proteins overlapped. In others they did not. Depletion of αEC showed that E-cadherin expression at the cell surface is not αEC–dependent, although we cannot rule out the possibility that some of it has gone after αEC depletion. Ectopic expression of E-cadherin in the blastula offered the opportunity to test this context dependence. We showed previously that E-cadherin can rescue the depletion of C-cadherin in the blastula [18]. However, we did not test whether this was αEC-dependent. We therefore depleted αEC in cultured oocytes, and subsequently injected them with E-cadherin-HA mRNA before fertilization. Animal caps were removed at the late blastula stage, and viewed *en face* under the confocal microscope. The results are shown in **Fig. 11**. E-cadherin did not rescue the loss of cell adhesion or the F-actin cytoskelton in the absence of maternal αEC in the blastocelic roof cells and these remained dissociated (**Fig. 11** **A, B, C**), although the E-cadherin protein was translated and was present at high levels in these embryos (**Fig. 11** **F**). However, when the superficial layer of cells in the animal caps was examined, in samples where all the deeper (blastocelic roof) cells had fallen off, E-cadherin was seen to be expressed at cell-cell contact sites and rescued the dissociation of αEC- depleted cells (**Fig. 11** **D,E**). Imaging the animal caps from the outside also showed the same result (data not shown). These results suggest that E-cadherin expression and function in cell adhesion is context-dependent. In the deep cells of the animal cap, E-cadherin required αEC, whereas in the superficial cells it did not.

## Discussion

The *Xenopus* embryo is a useful model system for this kind of study. It is an *in vivo* model in which the distribution and concentration of target proteins can be directly correlated with the phenotypic effects *in vivo*. The large size of its cells allows the visualization of cadherins and the actin skeleton at relatively low magnification. The ability to deplete individual target proteins, and replace them with either mutant forms, or related proteins, allows functional analysis of the differences between structurally related proteins, and of specific domains. We have used this model previously to show that the cadherins play a much larger role in morphogenesis than simply anchoring F-actin at the cell surface to stabilize cell contacts. They are also essential for F-actin assembly in the cell cortex of the early embryo [17], and different cadherins control different kinds of morphogenetic movement through the actin skeletons associated with them [18], [28]. In this paper we show that the α-catenins associated with cadherins also play different roles in morphogenesis, through unexpected differences in their interactions with specific cadherins. C-cadherin is the major cadherin of pre-gastrula stages, and remains co-expressed in the ectoderm with novel cadherins that appear at or after gastrulation. It requires αEC throughout early development for its expression on the cell surface. In these studies we were unable to distinguish between initial membrane presentation of C-cadherin and its stability on the cell surface. Various inhibitors of membrane turnover were tried, but did not give consistent results. Further insight into this will require further experiments. However, the control of C-cadherin expression was not principally at the level of protein translation or degradation, since the overall level of C-cadherin in the embryo was only modestly reduced in the absence of αEC.

Previous work showed that depletion of αEC caused a reduction in cell adhesion at the blastula stage [19]. At that time it was thought that the principal role of α-catenins was to provide a linkage between the cadherin complex, through binding to either β-catenin or plakoglobin, to the actin skeleton. Here we show that another role for C-cadherin binding to αEC is to present, or retain, C-cadherin in cell junctions. Replacement of the CBD by the C-terminal domain of αEC caused cadherin to be expressed on the surface without association with β-catenin, and bypassing any other proteins associated with the CBD. This suggests that the primary function of the CBD is to bind α-catenin and control cell surface expression of the cadherin.

The exact role played by αEC in this process is still unknown. It is possible that it may be involved in either targeting cadherin/catenin complexes to the cell surface or stabilizing cadherins in junctions by preventing endocytosis. αEC was recently shown to bind dynamitin and to regulate dynactin-based trafficking of lysosomes [29], suggesting that α-catenin could control the transport of cadherin/catenin complexes in *Xenopus*.

We have shown previously that all three major classical cadherins (C-,E-, and N-cadherin), in *Xenopus*, are expressed on the cell surface, and assemble F-actin, in pluripotent blastula cells lacking the endogenously expressed C-cadherin. However, by the neurula stage, in the embryonic ectoderm, they lose their ability to substitute for one another. The mechanism of this specialization is not known, although recent work suggested that the cadherin extracellular domain plays a role in regulating distribution through interactions with other cell adhesion molecules such as nectin [28]. In this work we show that specific interactions with α-catenins may also underlie this functional specificity of classical cadherins.

One of the most interesting questions that arises from this work is how cadherin-α-catenin interactions can be specific, when α-catenins bind, not directly to cadherins, but through β-catenin in each case. The answer to this will only come from a fuller understanding of how each component in the complex affects the binding of others. A number of proteins, in addition to actin and β-catenin, are known to bind α-catenin, including vinculin, α-actinin, ZO-1, afadin, formin, and Rho [10], [30]–[37], reviewed in [38]. However, it is not known whether binding confers any consequent specificity to α-catenin binding to different cadherins, or even whether these proteins bind to both αEC and αNC. In a complementary fashion, proteins recently found to bind cadherins, such as eplin [9], or nectin [28], may have consequent effects on specificity of α-catenin binding, since it is not known at present whether their interactions show any cadherin specificity.

In conclusion, it is becoming increasingly obvious from this and other studies that the cadherin-α-catenin interaction is not a simple linear link from cell surface cadherins to the actin cytoskeleton. α-catenins are responsible for maintaining cell surface expression of the cadherins, but in both a cadherin-specific, and context-dependent manner. They are also required for adhesive function of C- and N-cadherins, but probably not E-cadherin (at least in the non-neural ectoderm of *Xenopus*). In addition, as has been shown previously in the epidermis [24], αEC can also play a role in growth control in the embryo, but only of some tissues. In this study the E-cadherin-expressing non-neural ectoderm, but not the neural ectoderm nor the blastula, underwent massive expansion after αEC depletion. In addition, E-cadherin requires αEC for junctional localization in some tissues, but not others, suggesting that the functions of α-catenins can be context dependent. Further studies of α-catenin-binding proteins may elucidate the mechanism for this.

## Materials and Methods

### Ethics Statement

All *Xenopus laevis* experiments in this study have been conducted under protocol # 2D02014, approved by the Cincinnati Children’s Hospital Medical Research Foundation’s Institutional Animal Care and Use Committee.

### Oocytes and Embryos

All *Xenopus* animals used in this investigation were purchased from Nasco (Fort Akinson, WI). Host transfer experiments were done following the protocol described previously [14]. Stage VI oocytes were isolated from two year old female ovaries and cultured in Oocyte Culture Media (OCM). All anti sense oligos (AS oligos) were micro-injected into isolated oocytes in the equatorial region and cultured for at least 24 hours before microinjecting various mRNA constructs as described in the text. Only brown vital dye was used to color oocytes to prevent any background signals during confocal imaging of animal caps using fluorescent antibodies. In general, embryos were cultured in 0.1XMMR until the mid blastula stage and transfered to 1XMMR in special situations as described in the text. mRNA and morpholino injections to embryos were carried out in 2% ficoll solution.

### DNA Constructs and mRNA

C-cadherin mRNA used in this study were synthesized using the Sp6 mMESSAGE mMACHINE Kit (Ambion) using the same construct as previously described [13], [17]. nEαC construct was sub-cloned into pRN3 expression vector using the pBATEαC construct generously provided by Dr. Nagafuchi [39]. A two step ligation method was used. First, pRN3 expression vector was digested with Bgl-II and NotI restriction enzymes. pBATEαC construct was digested with Bgl-II and XbaI to release the insert containing the nEαC coding sequence. The Bgl-II sites of the purified insert band and the digested pRN3 construct was ligated in the first ligation step. The sticky NotI end from the pRN3 vector and the XbaI end from the insert were then filled in using Klenow DNA polymerase. A second ligation step was then performed to ligate the blunt ends and transformed into DH 5α cells. Insert-containing colonies were screened for and identified using restriction enzyme digest patterns and DNA sequencing. To make capped mRNA for micro-injection, nEαC-pRN3 construct was linearized using SfiI and and transcribed using T3 mMESSAGE mMACHINE Kit (Ambion). Myc-tagged human α-E-catenin cDNA was PCR amplified from α-catenin 2AB flag (gift from D.L. Rimm, Yale University) using PfuUltra Polymerase (Stratagene, La Jolla, CA) and cloned into the *Xenopus* expression vector pCS107, using EcoRI and NotI sites by Rebecca Daugherty (Northwestern University). Capped mRNA for microinjecting was synthesized by linearizing the constructs with Ascl and using the SP6 mMESSAGE mMACHINE Kit. The morpholino resistant α-N-catenin-HA construct was made by PCR amplifying the full length *Xenopus laevis* α-N-catenin (IMAGE clone: 7019675, Open biosystems), using the following primer pair: Left 5′-TCGAGCGGCCGCGCCACCATGTCTTCTGCC ACATCACCCATCATTCTCAAATGG-3′ ; Right 5′-TCGAAGGCCTTTAGA GGCTAGCATAATCAGGAACATCATACGGATAGAAT GAATCCATAGCTTT AAATTC-3′ and sub cloned into the NotI and StuI sites of the pCS107 expression vector. Capped mRNA was synthasized by linearizing the template with AscI and the SP6 mMESSAGE mMACHINE Kit. *Xenopus tropicalis* N-cadherin–Myc, and *Xenopus laevis* E-cadherin-HA mRNA were synthasized as previously described [18].

### Oligonucleotides

Anti-sense oligos (designated as AS throughout the text) used in this study are the following:

C-cadherin AS, 5′-C*C*T*CTCCAGCTCCCT*A*C*G-3′ [13], [17] was used at 2.4 ng per oocyte. α-catenin AS, 5′-A*G*T*CTCTCAACGGCT*A*G*T-3′ [19] was used at 10 ng per oocyte. * indicates a phosphorothioate modified bond. Antisense morpholino oligos (designated as MO throughout the text) used are, α-E-catenin morpholino: 5′-ATGTTTCCTGTATTGAGAGTCATGC-3′, α-N-catenin morpholino: 5′-GGCAGAAGACATGTTCCTCTATTG -3′ (GeneTools, LLC). A dose of 40 ng of α-E-catenin morpholino was injected into oocytes for maternal depletion experiments, and doses of 10–20 ng of either alpha catenin morpholino (per cell) was used for 2–8 cell stage embryonic injections.

### *In-situ* Probes

Wholemount In-situ’s for α-E-catenin, and α-N-catenin, were carried out using a standard *in-situ* protocol using double Dig labeled custom LNA mRNA detection probes (Exiqon). For α-N-catenin: 5DigN/ATGCACAACTGACACTACAAT/3DigN; for α-E-catenin: 5DigN/TACGCTCACCAGAAACCAGT/3DigN probes were used. Probes were resuspended in hybridization buffer to make 20 µM (40X) stock solutions, and were then hybridized at a 0.5 µM final concentration at 53°C. Samples were developed using BM Purple detection reagent (Roche) at 4°C. After the stainings were complete, samples were washed, and fixed in Bouin’s fixative overnight and bleached using a standard bleaching solution ( 1% H_2_O_2_, 5% Formamide, in 0.5X SSC), and a fluorescent light source to remove pigments.

### F-actin Staining and Immunostaining

Late blastula (Stage 9, [40]) animal caps were dissected and immediately fixed for 10 minutes in FG fixative as previously described by [17], washed for 30 minutes using PBSTw (PBS+0.1% Tween 20) and stained with Alexa-488 conjugated phalloidin (5 U/ml) for at least 4 hours in room temperature in PBSTw. Samples were then washed for two to three hours in PBSTw. For immunostaining of neurula stage embryos, whole embryos were fixed in 2%TCA for 2 hours at roomtemperature and then bisected and fixed for an additional one hour. For F-actin staining of neurula, embryos were bisected in 1XMMR and fixed in FG fixative for 15 minutes and washed for atleast 30 minutes in PBSTw. Samples were blocked in 10% Normal Goat Serum (NGS) for at least 1 hour and incubated in 4°C overnight with primary antibodies. All primary antibodies were incubated in 10% NGS. For C-cadherin, and E-cadherin immunostaining, we used the monoclonal antibodies 6B6 and 5D3 respectively (Developmental Studies, Hybridoma Bank, Iowa City) against *Xenopus* C-, and E-, cadherin at a 1∶200 dilution as previously described [17], [18]. For immunostaining of nEαC, a rat monoclonal antibody against mouse E-cadherin extracellular domain (ECCD2, generously provided by Dr. Takeichi) was used under the same fixation and washing conditions used for C-cadherin staining. For alpha-E-catenin staining, a rabbit ployclonal antibody against human alpha-E-catenin (Thermo Scientific, Pierce antibody Cat. # PA1-26445) was used in 2% TCA fixed tissues or FG fixed tissues at a 1∶200 dilution. For alpha-N-catenin immunostaining, a rat monoclonal antibody against chicken alpha-N-catenin (NCAT2, generously provided by Dr. Takeichi) was used at 1∶200 dilution under the same fixation and washing conditions used for C-cadherin staining. For β-catenin staining, a mouse monoclonal antibody against human β -catenin (E5) (Santa Cruz antibodies Cat. # Sc-7963) was used at 1∶200 dilution. After the primary antibody incubation samples were washed in PBSTw for 3 hours at room temperature. For E-, and C-, cadherins, DyLight-647 conjugated goat anti mouse secondary antibody (Jackson ImmunoResearch) was used at 1∶200 dilution. For α-E-catenin and GFP staining Alexa-488 conjugated goat anti rabbit secondary antibody (Jackson Immuno Research) was used at 1∶200 dilution. For nEαC , HA tag, and α-N-catenin, DyLight-647 conjugated goat anti rat secondary antibody was used at a 1∶200 dilution (Jackson Immuno Research). Samples were then washed in PBSTw for at least 2 hours in room temperature before imaging. For cleared ventral ectoderm samples, bisected and stained neurula were dehydrated and cleared in Murray’s clear (2 parts benzyl benzoate and 1 part benzyl alcohol). Atto-488 conjugated goat anti mouse secondary antibody (Sigma-Aldrich Cat.# 62197) was used at 1∶300 dilution for C-, and E-, cadherin primary antibody detection, and Alexa-647 conjugated goat anti rabbit secondary antibody (Invitrogen, Cat. # A-21245) was used at 1∶200 dilution for detecting α-E-catenin in cleared samples.

### Confocal Imaging

A Zeis LSM 510 inverted confocal microscope was used for imaging immunostained samples in this study. A LD C-Apochromat 40X/1.1 W korr UV-vis IR objective was used for visualizing both F-actin and C-cadherin staining under high magnification. Same imaging settings including the laser power level, gain, and pinhole size were used between all groups compared in an single experiment. Imaging conditions were set so that more than 90% of the imaged pixels were in the linear range in the control group of an experiment. All immunostaining pictures represent maximum intensity projections of Z-stacks. High resolution imaging of cleared samples and sections (**Fig. 1** **E, F, G, H** and **Fig. 10**) were done using a Nikon A1R si confocal microscope and a 100× Apo TIRF NA 1.49 oil objective. Images were aquired at nyquist limit using a 2× zoom in the center of the objective field. The 3D projections in **Fig. 1** **E, F, G, H** were created using the Nikon image analyser volume function. The 3D projections in **Fig. 10**, and **movies S3 and S4**, were created using Bitplane’s Imaris software.

### Quantification

For all quantifications, the Zeis LSM510 software was used to analyze individual projections of Z-stacks. F-actin levels of animal caps were quantified by taking measurements of the Alexa-488 conjugated phalloidin fluorescence level of whole animal caps and taking the average readings of 5–10 animal caps from a single experimental group as previously described in detail [17]. F-actin levels in neurula stage clones were qunatified by taking the average of individual cells as described previously [18]. Cadherin levels were quantified using the histogram function of the software by measuring the average pixel intensities across cell membranes [18]. Statistical significance was analyzed using the Student’s *t-*test. In figures, *indicates p<0.05, **p<0.01, ***p<0.001.

### Western Blotting

5 embryos or oocytes for each experimental group were frozen in dry ice and lysed using 50 µl of ice-cold PBSTx (PBS+1% Triton X-100) containing 1 mM PMSF and 0.01% PIC (Protease Inhibitor Complex, Sigma P8340). Samples were then cleared by a low speed centrifugation step (750× *g* for 10 minutes) and equal volumes of supernatants were removed and diluted in equal volumes of 4X sample buffer. Samples were then boiled for 5 minutes and 2 embryos equaling lysate (2/5 of final volume) was loaded in to a single lane of a 8% SDS PAGE gel and run for 2 ½ hours at 100 volts. Protein bands were then transferred into nitrocellulose membranes using the wet-transfer method and blocked in 5% non-fat milk. For C-cadherin staining, the 6B6 monoclonal antibody, and for E-cadherin, the 5D3 monoclonal antibody, were used at 1∶1000 and incubated at 4°C overnight. For α-tubulin, the DM 1A monoclonal antibody (Sigma, T-9026) was used at 1∶12000 dilution and incubated for no more than 3 hours in room temperature. For α-E-catenin staining, the rabbit ployclonal antibody against human α-E-catenin (Thermo Scientific, Pierce antibody Cat. # PA1-26445) was used at 1∶1000 and incubated at 4°C overnight. For β-catenin staining, a mouse monoclonal antibody aginst human β-catenin (E5) (Santa Cruz antibodies Cat. # Sc-7963) was used at 1∶2500 dilution. For HA staining, the rat anti-HA monoclonal antibody 3F10 (Roche) was used at 1∶5000 dilution. For Myc tag staining, a rabbit polyclonal anti-Myc antibody (Cell signaling, cat.#2272S) was used at 1∶5000 dilution. After washing the membranes for at least 1hour in PBSTw, HRP conjugated goat anti mouse, or anti rabbit, secondary antibodies (Jackson Immuno Research) were used at 1∶5000 dilutions and incubated for 1 hour at room temperature. ECL western blot developing solutions (Amersham) were used to detect the secondary antibodies after a 1 hour PBS wash step. Membranes were then exposed to X-ray films accordingly to obtain unsaturated bands and the films developed using a standard western blot-developing machine. For stripping and reprobing membranes, Onemiute westernblot stripping buffer (GM Biosciences) was used as directed.

### Co-Immunoprecipitations

For each immunoprecipitation (IP), at least 50 embryos were used per sample. All immunoprecipitation experiments were carried out using st. 19 embryos and protein-G conjugated agarose beads (Roche), as previously described [41], [42] with the exception of 1% IGEPAL CA-630 (NP-40 substitute, Sigma-Aldrich), as the detergent instead of using Triton X-100. For HA tag IP, the high affinity anti-HA rat monoclonal antibody 3F10 (Roche, Cat.# 11867431001), for Myc tag IP, a rabbit polyclonal anti-Myc antibody (Cell signaling, cat.#2272S) was used. For E-cadherin IP, the mouse monoclonal antiboody aginst *Xenopus* E-cadherin 5D3 (Developmental Studies, Hybridoma Bank, Iowa City) was used.

## Supporting Information

Movie S1 **Cyst formation in α-E-catenin depleted embryos.** 10 ng of αEC morpholino was injected to one cell at the two cell stage. Embryos were cultured in1XMMR following gastrulation. Photographs for the timelpase were taken at 5 minute intervals.
(MOV)

Movie S2 **Blockade and rescue of neurulation movements in α-N-catenin-depleted embryos.** Embryo on the left is an uninjected control. Middle embryo was injected with 20 ng of αNC morpholino in to each dorsal animal cell of the 8 cell stage, the embryo on right was injected with a morpholino-resistant αNC mRNA following the injection of the morpholino.
(MOV)

Movie S3 **3D-image projection of a cleared, E-cadherin and α-E-catenin stained non-neural ectoderm, aquired by high resolution confocal microscopy.** E-cadherin is stained green and αEC is stained red.
(MOV)

Movie S4 **3D-image projection of a cleared, C-cadherin and α-E-catenin stained non-neural ectoderm, aquired by high resolution confocal microscopy.** C-cadherin is stained green and αEC is stained red.
(MOV)
